# Changing etiologies and outcome of liver failure in Southwest China

**DOI:** 10.1186/s12985-016-0536-0

**Published:** 2016-06-04

**Authors:** Gui-Juan Xie, Hui-Yan Zhang, Qing Chen, Hui-Min Liu, Jian-Ping You, Sha Yang, Qing Mao, Xu-Qing Zhang

**Affiliations:** Department of Infectious Diseases, Southwest Hospital, Third Military Medical University, Chongqing, 400038 P.R. China

**Keywords:** Viral hepatitis, Liver failure, Etiology, Prognosis, Antiviral therapy related liver failure

## Abstract

**Background:**

The prognosis of liver failure depends greatly on the underlying cause, and there were few data about the prognosis, etiologies or trigger factors of liver failure in China based on long-term and large samples cohorts.

**Methods:**

We screened out 3171 liver failure cases from 25467 patients hospitalized in our department between 2000 and 2012 according to Chinese criteria, and determined their etiologies and prognosis.

**Results:**

97.3 % cases were associated with at least one of 25 identified factors. The 3 leading etiologies were HBV (91.6 %), alcohol (18.1 %) and antiviral therapy (AVT) related hepatitis B flares (6.7 %). Acute-on-chronic liver failure (ACLF) accounted for 92.1 % of all cases. 96.5 % ACLF cases were associated with HBV, in which the percentage of AVT related flares increased from 0 % in 2000 up to 11.5 % in 2012, and hepatitis virus superinfection declined from peak 19.3 % in 2002 down to 2.5 % in 2012. Three-month spontaneous survival (SS) rate of 3171 cases was 31.4 %, but improved from 17.4 % in 2000 up to 40.4 % in 2012. SS was significantly different among various etiological groups (*P* = 0.000). In HBV related liver failure aged 25 to 54 years, males accounted for 87.6 %, and had a progressively decreased SS with increasing age. From 25 to 54 years, SS was lower in male than in female HBV related liver failure, and having significant difference in cases of ages 40 to 44 years (27.6 % versus 50.9 %, *P* = 0.001).

**Conclusion:**

Etiologies of liver failure were numerous and varied in southwest China. HBV was the most leading cause of liver failure, especially in ACLF. AVT related flares had become the third leading cause of ACLF. The prognosis of liver failure remained poor, but had markedly improved in recently 3 years. Middle-aged male HBsAg carriers had an extremely higher risk for liver failure and worse prognosis compared to female.

1. Etiologies of liver failure were numerous and varied in southwest China. HBV infection is the main cause of liver failure in southwest China, especially the major cause of ACLF. Antiviral related liver failure, especially the NUCs withdrawal induced ACLF were extremely increased, which has replaced the superinfection as the third important cause of HBV-ACLF.
2. The prognosis of liver failure is still poor, but the spontaneous survival rate showed a trend of steady rise in recent years. The prognosis of patients with liver failure caused by different causes also exists certain difference, the more damage factors bulls the worse prognosis.
3. The prognosis of the HBV and HCV reactivation induced by the steroids was poor.Interferon treatment of CHB in ACLF although rare, but should be taken into consideration seriously.
4. Patients with liver failure caused by different etiologies showed larger differences of gender and age distribution. Gender and age are the important factors with the occurrence and prognosis of HBV-ACLF.

## Background

Liver failure is a serious acute or chronic liver insufficiency induced by a variety of causes. However, there are high diversity on the definition, classification and criteria of liver failure in different countries [1]. In China, liver failure is classified into acute liver failure (ALF), sub-acute liver failure (SALF), acute-on-chronic liver failure (ACLF) and chronic liver failure [2]. ALF or SALF is a clinical syndrome characterized by acute liver decompensation developed in the absence of pre-existing liver disease [3]. ACLF is another clinical type of acute liver decompensation resulting from a precipitating event (acute insult) in patients with previously compensated liver disease [4]. Acute liver decompensation may be reversible if the trigger factor is treated, but associated with high short-term mortality in ALF, SALF and ACLF. The main causal agents of liver failure show wide geographical variation, and depend on the prevalent hepatotropic virus infections and patterns of drug use. Otherwise, the prognosis of liver failure depends greatly on the underlying cause. Therefore, it is important to perform a survey on the changing etiologies and trigger factors of liver failure in China.

Numerous reports on the etiology of ALF and SALF have been published in different countries. In USA, acetaminophen toxicity was the most common cause of ALF [5]. Zhao reported that drug toxicity, indeterminate and viral hepatitis were the main causes of ALF in China [6]. However, there are few epidemiological and etiological studies regarding ACLF. China is a region of high prevalence of hepatitis B virus (HBV) infection. HBV associated ACLF is the most common clinical type of liver failure in China [2]. The acute events that trigger the onset of liver failure in HBV carriers show wide geographical variation and vary in different periods [4]. Some previous studies based on small samples cohorts showed that hepatitis E virus (HEV) infection is a leading trigger factor of ACLF in Bangladesh, India and southeast China [7–9]. However, there were few data about the prognosis, etiologies or trigger factors of liver failure in China based on long-term and large samples cohorts. Otherwise, it is also not consistent about the association of prognosis with etiologies, sex and age in previously published data. In this study, we performed an extensive investigation on 3171 liver failure patients admitted in our department from 2000 to 2012, in order to clarify the prognosis and epidemiological characteristics of liver failure in China, which included transplant-free or spontaneous survival (SS) rate, etiologies, sex, age and their association.

## Methods

### Patients collection

**Fig. 1:**
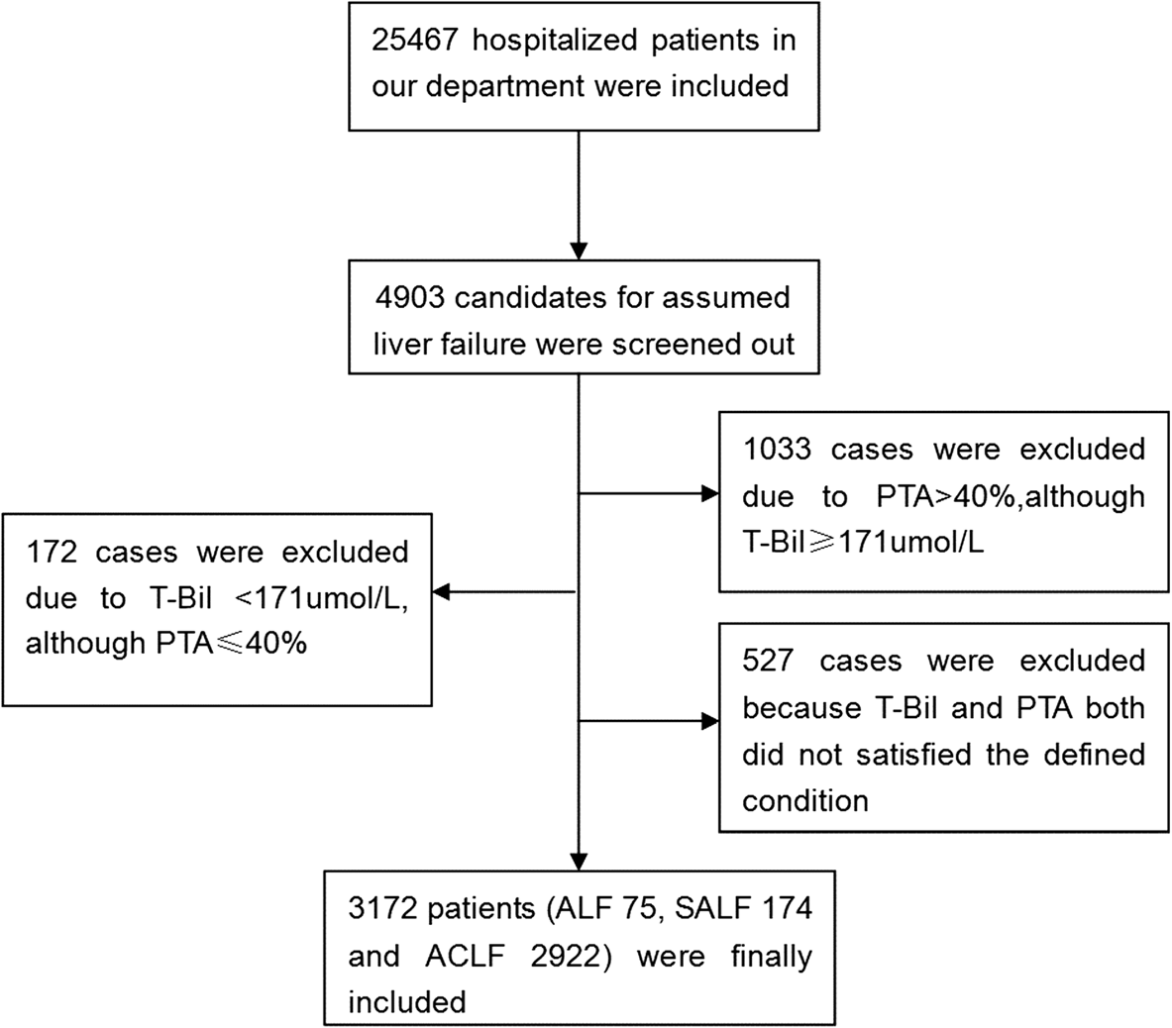
Enrollment of patients in this study. Abbreviation: ALF, acute liver failure; SALF, sub-acute liver failure; ACLF, acute-on-chronic liver failure; PTA, prothrombin activity; TBil, total bilirubin

Figure 1 shows the enrollment of patients in this retrospective study. Firstly, we made a search in computerized diagnoses hospital registry for all patients admitted to department of infectious diseases, Southwest hospital of Chongqing, China from January 2000 to December 2012. A total of 25467 hospitalized patients were indexed during the 13 years period. Secondly, we screened out 4903 candidate cases for assumed liver failure according to the diagnostic records obtained from computerized diagnoses hospital registry, which included 5 cases of fulminant hepatic failure, 71 cases of liver failure, 52 cases of ALF, 48 cases of SALF, 62 cases of ACLF, 71 cases of fulminant viral hepatitis, 56 cases of subfulminant viral hepatitis, 4135 cases of chronic severe hepatitis B, 7 cases of chronic severe hepatitis C, 6 cases of severe acute hepatitis B, 8 cases of severe alcoholic hepatitis, 6 cases of severe drug hepatitis, 325 cases of severe chronic hepatitis B, 48 cases of Wilson’s disease and 13 cases of acute fatty liver of pregnancy (AFLP). Thirdly, each of 4903 cases with assumed liver failure was again determined to be whether or not fulfilled the diagnostic criteria of ALF, SALF and ACLF in China according to the following clinical data obtained from the electronic medical records, which included the levels of prothrombin activity (PTA) and serum total bilirubin (T-Bil), the length of illness, a history of underlying chronic liver disease, the grade of hepatic encephalopathy (HE) and the time from onset of illness to development of HE. Inclusion criteria for ALF included: ① serum T-Bil≧10 mg/dL or an increased T-Bil/d ≧ 1 mg/dL; ② PTA ≤40 %; ③ the occurrence of at least gradeII HE within 2 weeks of illness onset; ④ without pre-existing liver disease. Inclusion criteria for SALF included: ① serum T-Bil≧10 mg/dL; ②PTA ≤40 %; ③the length of illness <26 weeks with or without HE; ④ without pre-existing liver disease. Inclusion criteria for ACLF was an onset of acute liver decompensation within 12 weeks in a patient with previously diagnosed or undiagnosed chronic liver disease, and included the following evidences: ① serum T-Bil≧10 mg/dL; ②PTA ≤40 %. Finally, 3171 patients were enrolled in this study based on the above inclusion criteria, which included 75 cases of ALF, 174 cases of SALF and 2922 cases of ACLF. Among 3171 patients with liver failure, 3118 (98.33 %) cases were from Southwest areas of China, which included Chongqing (60.99 %), Sichuan (32.17 %), Guizhou (4.73 %), Yunnan province (0.28 %), Tibet (0.09 %) and Guangxi (0.06 %). Another 53 cases (1.67 %) were from other areas of China, which included Liaoning, Jilin, Anhui, Jiangsu, Jiangxi, Zhejiang, Fujian, Guangdong, Hainan, Hubei, Hunan, Shanxi, Henan, Shandong province and Xinjiang. After informed consent was obtained from the next of kin, detailed prospective clinical and laboratory data were collected in an anonymous fashion on admission to our hospital. The protocol was approved by the Ethics Committee of Southwest Hospital, Chongqing, China.

### Defining the etiologies and trigger factors of liver failure

**Viral infection** was defined based on serum markers for hepatitis A-E, human immunodeficiency virus (HIV), herpes simplex virus (HSV), Epstein-Barr virus (EBV) and cytomegalovirus (CMV). All 3171 patients were tested for serum anti-HAV IgM, HBsAg and HBV DNA, anti-HCV, HDAg and anti-HDV, anti-HEV IgM, and anti-HIV. Only patients who did not suffer from hepatitis A-E were tested for IgM antibody of HSV, EBV and CMV. **Autoimmune hepatitis** (**AIH**) was diagnosed based on descriptive clinical criteria reported by the International Autoimmune Hepatitis Group [10]. **Alcoholic liver disease** (**ALD**) was considered for patients who had taken alcohol at least 3 years, and a regular average consumption of ethanol >40 g/day for women and >60 g/day for men in this article [11]. **Drug induced liver injury** (**DILI**) was considered for patients who had taken any hepatotoxic drugs or Chinese herbs within 6 months before this illness onset. **Wilson**’**s disease** was diagnosed according to the criteria recommended by European Association for the Study of the Liver. The clinical diagnosis for other diseases was obtained from the electronic medical records, included hyperthyroidism, schistosomiasis japonica, AFLP, acute pancreatitis, septic cholangitis and tumors. The etiology of liver failure was considered to be indeterminate if no diagnostic clues were obtained from the patient’s history, serologic markers for HAV, HBV, HCV, HEV, CMV, EBV, HSV, HIV, anti-smooth-muscle or antinuclear antibodies and ceruloplasmin (or other signs of Wilsons disease). Trigger factors of liver failure included the following events: ① nucleos(t)ide analogues (NUCs) withdrawal, which was defined as HBV reactivation and abnormal liver function test within 52 weeks of withdrawal from NUCs therapy among patients who received at least 12 weeks NUC therapy; ② resistance to NUCs; ③ steroids use induced HBV reactivation; ④ interferon (IFN) therapy induced severe acute exacerbation (SAE) of chronic hepatitis B (CHB); ⑤ other factors, such as surgery and pregnancy. Bacterial infection was not defined as a trigger factor in HBV associated liver failure because that this infection was not identified to be existed prior to onset of liver failure or secondary to liver failure. We defined ACLF induced by NUCs withdrawal, resistance to NUCs and IFN therapy as antiviral therapy related ACLF (AVT-ACLF).

### Statistical analysis

For processing the data, Microsoft Office Excel 2010(Microsoft, Redmond, WA) and IBM SPSS Statistics (IBM, Armonk, NY) were used. The data are shown in medians and interquartile ranges. Continuous variables were summarized as mean ± standard deviation (SD), median (range), or frequency (in percent). Categorical variables were compared with the chi-square test and Fisher’s exact test. A p value of <0.05 was considered statistically significant.

## Results

### Distribution and trend of etiologies for liver failure

In this study, 3171 patients were enrolled to be analyzed for etiologies of liver failure, in which 3086 (97.3 %) cases were identified to be associated with at least one of several causal agents for hepatic injury, and 85 (2.7 %) cases were indeterminate etiology. A total of 25 precipitating factors were identified in 3171 patients, in which the 5 leading causes were HBV (91.6 %), alcohol (18.1 %), antiviral therapy (AVT) related hepatitis flares (6.7 %), drugs (5.4 %) and HEV (3.2 %), shown in Table 1. Among 171 patient with DILI, 107 (62.6 %) had received antituberculosis drugs, 41 (24.0 %) had received Chinese herbs and 23 (13.5 %) had received other drugs which included nonsteroidal anti-inflammatories drugs (3.5 %), acetaminophen (2.3 %), antibiotics (1.8 %), leflunomide (1.8 %), statins (1.2 %), methimazole (1.2 %), anticancer drug (0.6 %), cyclosporine A (0.6 %) and chlorpromazine (0.6 %). Among 164 ACLF induced by withdrawal of NUCs, the percentage of cases after the withdrawal from lamivudine was 53.7 %, adefovir 26.8 %, entecavir 9.8 %, telbivudine 4.9 %, lamivudine and adefovir 4.9 %. Among 37 patients with HBV resistance to NUCs, the percentage of cases with resistance to lamivudine was 67.6 %, adefovir 16.2 %, telbivudine 8.1 %, adefovir and lamivudine 5.4 %, adefovir and entecavir 2.7 %.

**Table 1:** The etiologies of 3171 patients with liver failure

Etiologies	Frequency (%)		Etiologies	Frequency (%)	
HBV	2906	(91.64 %)	Hyperthyroidism	21	(0.66 %)
HEV	102	(3.22 %)	Tumor	21	(0.66 %)
HDV	74	(2.33 %)	AIH	19	(0.60 %)
HAV	27	(0.85 %)	Pregnancy	16	(0.50 %)
HCV	23	(0.73 %)	Acute pancreatitis	10	(0.32 %)
HIV	12	(0.38 %)	AFLP	8	(0.25 %)
CMV^a^	3		Wilson’s disease	6	(0.19 %)
EBV^a^	2		Schistosomiasis japonica	2	(0.06 %)
HSV^a^	2		Septic cholangitis	2	(0.06 %)
Alcohol	573	(18.07 %)	Indeterminate	85	(2.68 %)
Medicinal factor	452	(14.25 %)			
Drugs^b^	171	(5.39 %)			
Withdrawal of NUCs	164	(5.17 %)			
Steroids use	63	(1.99 %)			
Resistance to NUCs	37	(1.17 %)			
IFN therapy	12	(0.38 %)			
Surgery	7	(0.22 %)			

^a^Only patients who were negative for HAV, HBV, HCV, HDV and HEV were tested for serum IgM antibody of cytomegalovirus (CMV), Epstein-Barr virus (EBV) and herpes simplex virus (HSV). ^b^included 2 cases of steroids use

**Fig. 2:**
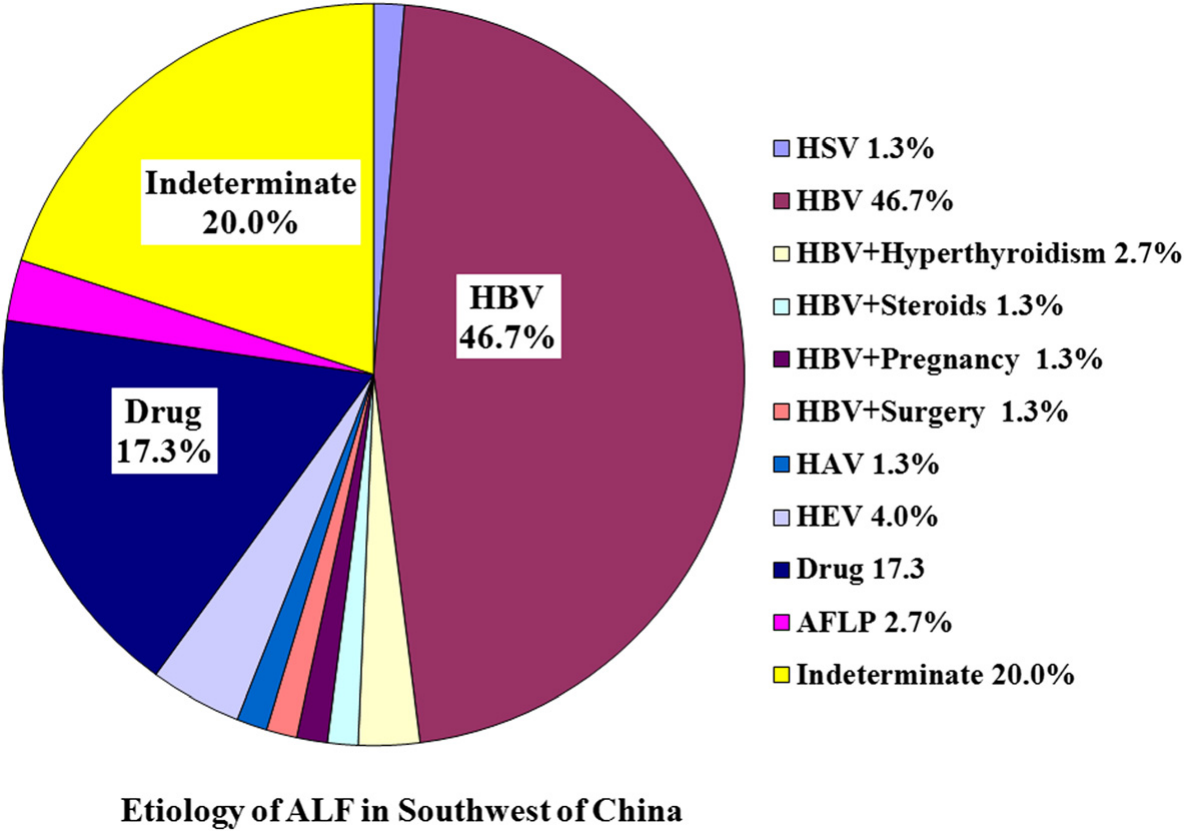
Distribution of etiologies of liver failure in ALF Southwest China

**Fig. 3:**
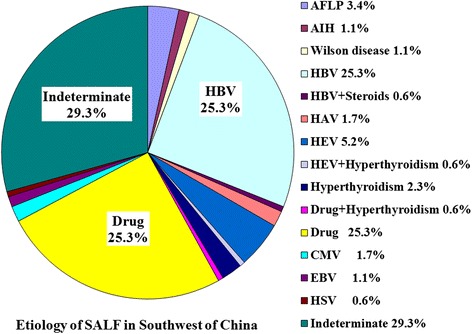
Distribution of etiologies of liver failure in SALF Southwest China

**Fig. 4:**
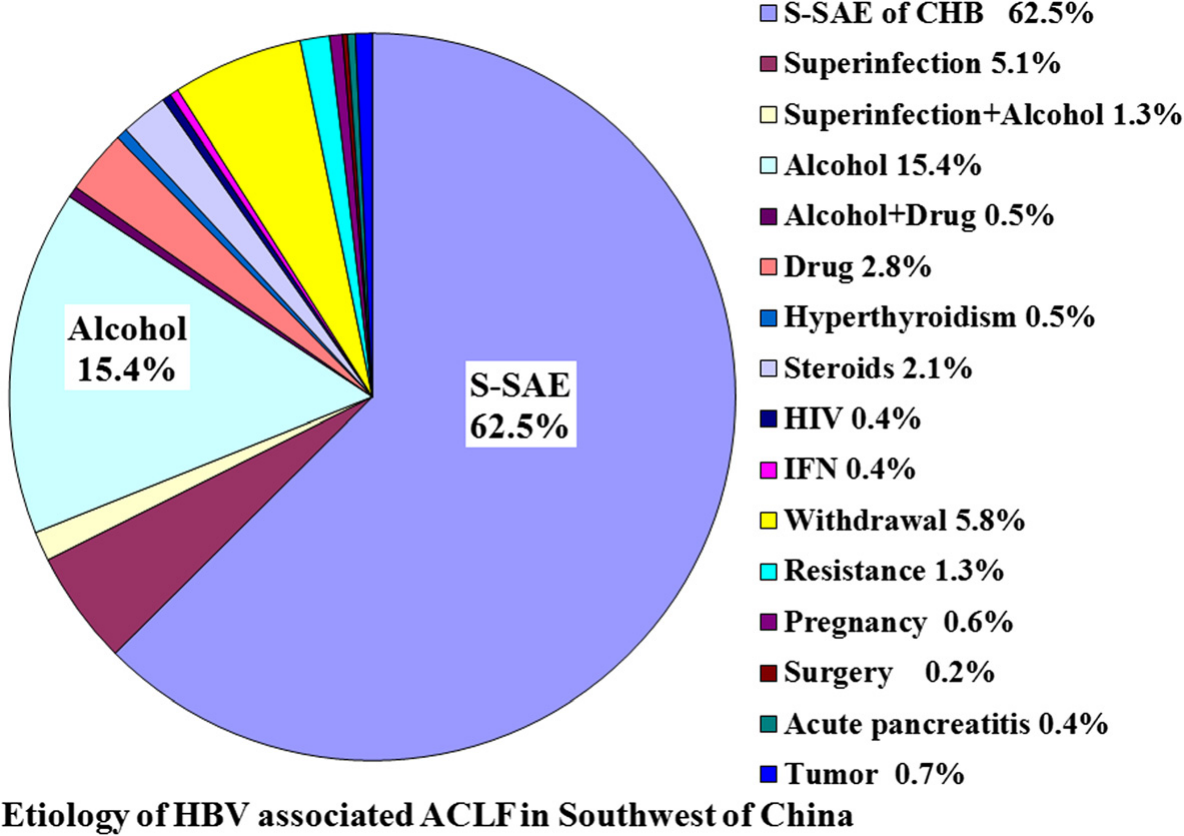
Distribution of etiologies of HBV related liver failure in ACLF Southwest China

The distribution of etiologies showed wide variation in different clinical type of liver failure. The 3 leading causes were single HBV infection (46.7 %), indeterminate (20.0 %) and DILI (17.3 %) for ALF (Fig. 2), but being indeterminate (29.3 %), single HBV infection (25.3 %) and DILI (25.3 %) for SALF (Fig. 3). Among 2922 patients with ACLF, 2821 (96.5 %) cases were associated with HBV infection, but only 101 (3.5 %) cases being non-HBV associated ACLF. Among 2821 patients with HBV associated ACLF, 1057 (37.5 %) cases had more than one precipitating factor, in which the 3 leading trigger factors were alcohol, superinfection and NUCs withdrawal (Fig. 4). The percentages of patients with at least two precipitating factors were significantly higher in ACLF (36.7 %, 1071/2922) than those in ALF (6.7 %, 5/75) and SALF (1.7 %, 3/174), *P* = 0.000.

**Fig. 5:**
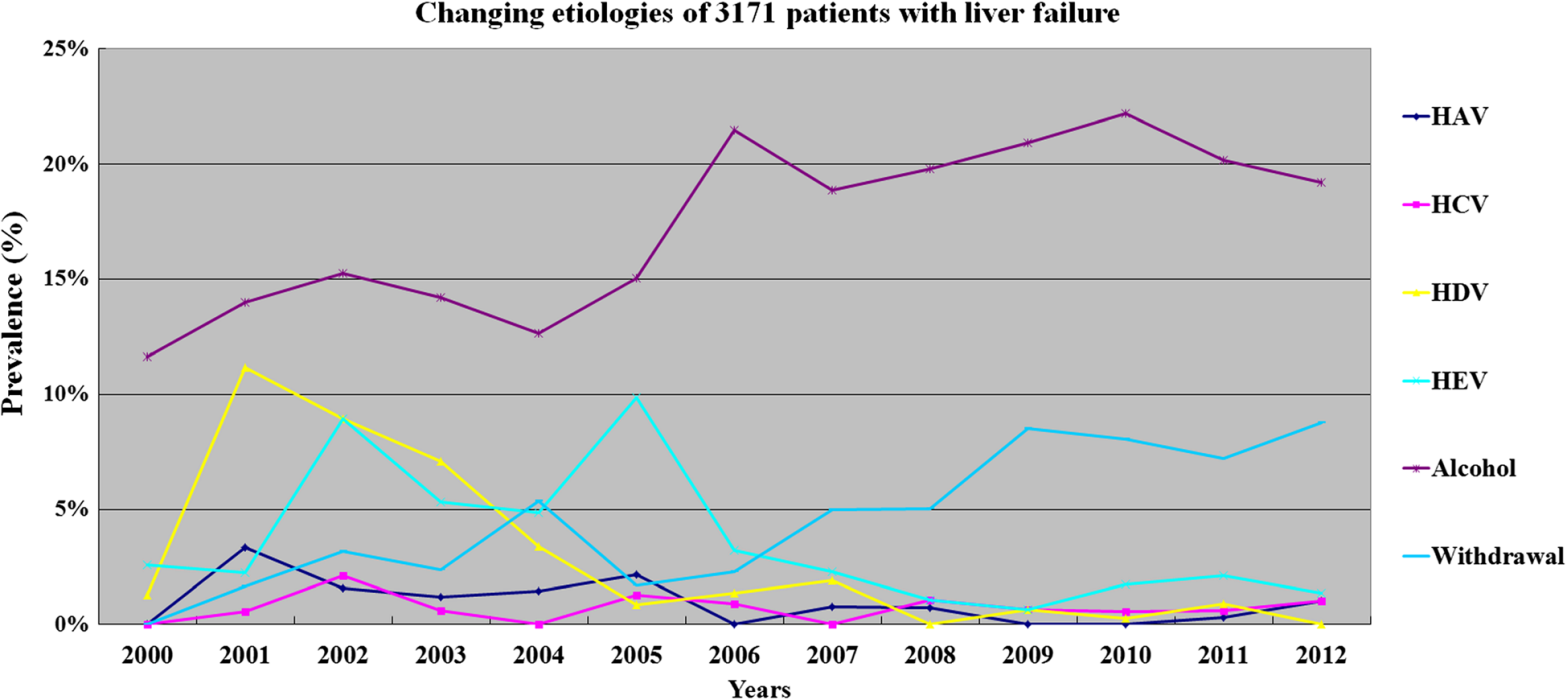
Changing common etiologies or trigger factors liver failure in Southwest China from 2000 to 2012

**Fig. 6:**
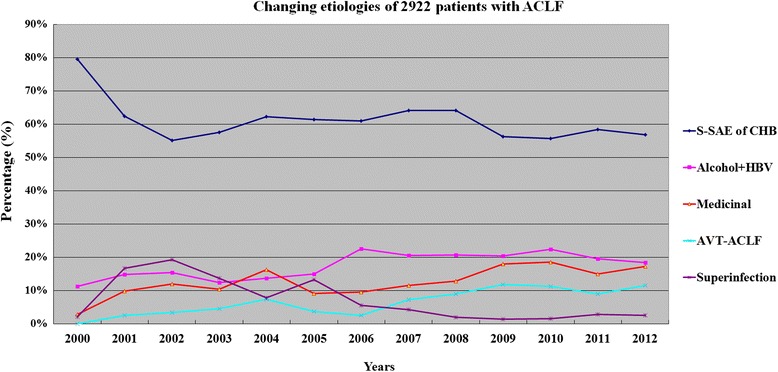
Changing common etiologies or trigger factors liver failure in Southwest China from 2000 to 2012. ALD, alcoholic liver disease; AVT-ACLF, antiviral therapy related ACLF; NUCs, nucleos (t)ide analogues; CHB, chronic hepatitis B; S-SAE, spontaneous SAE of CHB

The distribution of etiologies for liver failure also showed wide variation during the 13 years periods. Figures 5–6 showed the trends of common etiologies. The etiologies with a declined trend included HAV, HCV, HDV, HEV infection, while those of having an increased trend included ALD, NUCs withdrawal and resistance to NUCs. For ACLF, the 3 leading etiologies were spontaneous SAE of CHB (58.2 %, 286/491), hepatotropic viruses superinfection (17.1 %, 82/491) and HBV plus alcohol (14.3 %, 70/491) between 2001 and 2003, but were spontaneous SAE of CHB (58.9 %, 989/1678), HBV plus alcohol (20.3 %, 341/1678) and medicinal factors (15.7 %, 264/1678) including 170 (10.1 %) cases of AVT-ACLF between 2007 and 2012. Cases induced by hepatotropic viruses superinfection and HBV plus drugs only accounted for 2.4 % and 3.0 % of all 1678 ACLF patients between 2007 and 2012.

### Demographical characteristics of patients with liver failure

Demographical characteristics of liver failure in different clinical types and etiologies are depicted in Table 2. The ratio of male to female was 5.6:1 in ACLF, which was significantly higher than that in ALF (1.3:1) and SALF (0.9:1), *P* = 0.000. Patients with ACLF tended to be older than those with SALF and ALF (P = 0.000). There were no significant difference with age (*P* = 0.122) and sex (*P* = 0.269) between SALF and ALF. The ratio of male to female was 5.9:1 in HBV associated liver failure, which was significantly higher than that (0.9:1) in non-HBV associated liver failure (*P* = 0.000), but having no significant difference with age between two groups (*P* = 0.258). Patients with liver failure induced by Chinese herbs tended to be older (*P* = 0.001) and more likely to be female (*P* = 0.000) than those with liver failure induced by antituberculosis drugs. Patients with indeterminate liver failure tended to be younger (*P* = 0.015) and more likely to be female (*P* = 0.000) than those of HBV associated liver failure. In HBV associated liver failure, male patients (41.5 ± 11.3 years) tended to be younger than female (45.3 ± 13.0 years) cases (*P* = 0.000). 81.1 % patients with HBV related liver failure were aged 25–54 years in male, while only 67.2 % cases were aged 25–54 years in female, the ratio of male to female reached 7.1:1 (Table 3).

**Table 2:** Demographical characteristics of patients with liver failure

	Sex (%)			Age	
	Male	Female	*P* value	(years)	*P* value
All cases (*n* = 3171)	82.18 %	17.82 %		41.9 ± 12.4 (0.5–83)	
Clinical types					
ALF (*n* = 75)^a^	56.00 %	44.00 %	0.000	33.7 ± 17.5 (0.5–76)	0.000
SALF (*n* = 174)^a^	47.70 %	52.30 %	0.000	37.4 ± 17.4 (3–79)	0.000
ACLF (*n* = 2922)	84.90 %	15.10 %		42.4 ± 11.7 (8–83)	
HBV (*n* = 2906)	85.40 %	14.60 %		42.0 ± 11.7 (8–83)	
non-HBV (*n* = 265)^b^	47.20 %	52.80 %	0.000	40.7 ± 18.4 (0.5–79)	0.258
ALD (*n* = 573)	98.40 %	1.60 %	0.000	46.0 ± 10.3 (21–77)	0.000
DILI (*n* = 171)	69.60 %	30.40 %	0.000	41.7 ± 14.7 (4–75)	0.658
Antituberculosis (*n* = 107)	85.00 %	15.00 %		39.1 ± 12.2 (16–72)	
Chinese herbs (*n* = 41)&	36.60 %	63.40 %	0.000	48.6 ± 14.9 (11–72)	0.001
HAV (*n* = 27)	85.20 %	14.80 %	0.570	36.4 ± 15.2 (3–75)	0.033
HEV (*n* = 102)	89.20 %	10.80 %	0.167	42.2 ± 13.9 (17–81)	0.419
HDV (*n* = 74)	86.50 %	13.50 %	0.478	43.8 ± 11.7 (14–68)	0.084
HCV (*n* = 23)	87.00 %	13.00 %	0.563	38.4 ± 10.4 (24–64)	0.116
Withdrawal (*n* = 164)	84.30 %	15.70 %	0.506	42.5 ± 10.4 (20–71)	0.363
Resistance (*n* = 37)	83.80 %	16.20 %	0.461	42.4 ± 9.8 (13–63)	0.400
Steroids use (*n* = 63)	74.60 %	25.40 %	0.018	46.4 ± 12.5 (22–69)	0.005
Indeterminate (*n* = 85)^b^	37.60 %	62.40 %	0.000	38.8 ± 20.0 (0.5–79)	0.015

The results are shown as median and range

*ALF* acute liver failure, *SALF* sub-acute liver failure, *ACLF* acute-on-chronic liver failure, *ALD* Alcoholic liver disease, *DILI* Drug induced liver injury

^a^ALF, SALF VS ACLF;^b^ Non-HBV VS HBV; & Chinese herbs VS Antituberculosis

**Table 3:** The distribution of sex, age and etiologies, and their association with outcome in HBV related liver failure

	N of cases		Distribution		M/F	SS rates			ALD	Medicinal factors		Superinfection	
Age	Male	Female	Male	Female	ratio	Male	Female	*P* value	Male	Male	Female	Male	Female
<15	8	2	0.3 %	0.5 %	4.0	50.0 %	50.0 %	0.326	0.0 %	12.5 %	0.0 %	0.0 %	50.0 %
15–19	30	6	1.2 %	1.4 %	5.0	53.3 %	33.3 %	0.584	0.0 %	0.0 %	0.0 %	6.7 %	0.0 %
20–24	84	15	3.4 %	3.5 %	5.6	63.1 %	46.7 %	0.077	4.8 %	13.1 %	26.7 %	6.0 %	6.7 %
25–29	188	28	7.6 %	6.6 %	6.7	42.0 %	46.4 %	0.686	7.4 %	11.2 %	3.6 %	8.5 %	0.0 %
30–34	359	39	14.5 %	9.2 %	9.2	41.8 %	51.3 %	0.307	12.0 %	11.1 %	7.7 %	6.7 %	15.4 %
35–39	525	66	21.2 %	15.6 %	8.0	30.3 %	39.4 %	0.159	17.7 %	11.0 %	9.1 %	5.5 %	6.1 %
40–44	427	57	17.2 %	13.4 %	7.5	27.6 %	50.9 %	0.001	24.1 %	13.8 %	10.5 %	4.4 %	1.8 %
45–49	307	43	12.4 %	10.1 %	7.1	26.7 %	39.5 %	0.103	29.6 %	18.2 %	16.3 %	6.8 %	2.3 %
50–54	207	52	8.3 %	12.3 %	4.0	21.7 %	30.8 %	0.201	32.4 %	10.1 %	21.2 %	9.7 %	0.0 %
55–59	156	55	6.3 %	13.0 %	2.8	21.8 %	14.5 %	0.175	31.4 %	14.7 %	20.0 %	8.3 %	5.5 %
60–64	101	37	4.1 %	8.7 %	2.7	18.8 %	24.3 %	0.811	34.7 %	14.9 %	13.5 %	5.0 %	5.4 %
≧65	90	24	3.6 %	5.7 %	3.8	13.3 %	8.3 %	0.730	28.9 %	11.1 %	8.3 %	6.7 %	4.2 %
Total	2482	424	100.0 %	100.0 %	5.9	31.1 %	35.4 %		21.2 %	12.7 %	13.2 %	6.4 %	4.7 %

*SS* spontaneous survival, *ALD* Alcoholic liver disease

*P* value: SS in male versus in female

### Prognosis of liver failure

In this study, only 66 (2.1 %) patients received a liver transplant, and 45 (68.2 %) survived within 1 year of post-transplant. During the 13 years, the average 3-month SS rates of liver failure were 31.4 % (996 of 3171), but showed a gradually increased trend (Table 4). SS rates were 41.1 % (401/976) in 3 years between 2010 and 2012, which were significantly higher than 32.4 % (344/1063) in 4 years between 2006 and 2009 (*P* = 0.000), and 22.2 % (251/1132) in 6 years between 2000 and 2005 (*P* = 0.000).

**Table 4:** Spontaneous survival (SS) rates of patients with liver failure

	All patients		Clinical types			Etiologies	
Years	N	SS	ALF	SALF	ACLF	non-HBV	HBV
2000	155	17.4 %	0.0 %	0.0 %	19.0 %	0.0 %	19.0 %
2001	179	22.9 %	0.0 %	0.0 %	25.3 %	0.0 %	24.6 %
2002	190	22.1 %	0.0 %	22.2 %	22.7 %	8.3 %	23.0 %
2003	169	27.2 %	40.0 %	27.3 %	26.8 %	9.1 %	28.5 %
2004	206	25.7 %	33.3 %	0.0 %	26.2 %	20.0 %	26.2 %
2005	233	18.0 %	16.7 %	28.6 %	17.7 %	18.8 %	18.0 %
2006	219	33.3 %	0.0 %	45.5 %	34.0 %	35.3 %	33.2 %
2007	260	33.1 %	0.0 %	28.6 %	34.2 %	33.3 %	33.1 %
2008	278	31.7 %	0.0 %	41.2 %	31.6 %	30.0 %	31.8 %
2009	306	31.7 %	25.0 %	26.3 %	32.3 %	35.1 %	31.2 %
2010	347	42.9 %	40.0 %	42.1 %	43.1 %	47.4 %	42.4 %
2011	332	39.8 %	50.0 %	29.4 %	40.3 %	36.4 %	40.1 %
2012	297	40.4 %	0.0 %	47.1 %	40.3 %	26.9 %	41.7 %
Total	3171	31.4 %	17.3 %	29.3 %	31.9 %	28.3 %	31.7 %

SS rate of ALF was 17.3 % (13/75), which was significantly lower than 29.3 % (51/174) of SALF and 31.9 % (932/2922) of ACLF (*P* = 0.047, 0.007). In SALF and ACLF group, SS rates of patients with HE were 6.3 % (6/95) and 8.0 % (100/1245), which were significantly lower than 57.0 % (45/79) and 49.6 % (832/1677) of patients without HE (*P* = 0.000). In fact, SS rate was significantly higher in ALF (17.3 %) than in SALF (6.3 %) and ACLF (8.0 %) with HE (*P* = 0.028, 0.010).

**Fig. 7:**
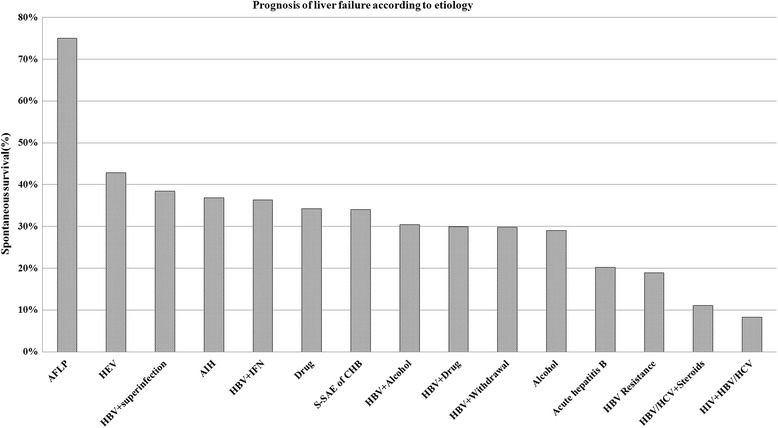
The outcome according to etiology amongst the different etiological groups of liver failure

Figure 7 showed SS rates of patients according to the etiology amongst the major etiological groups of liver failure. There were significant difference with SS rates among different etiological groups (*P* = 0.000). In HBV associated ACLF, SS rate of patients induced by alcohol, drugs, NUCs withdrawal and resistance to NUCs was 29.1 % (223/766), which was significantly lower than those of spontaneous SAE (34.1 %, 601/1764) and hepatotropic viruses superinfection (38.5 %, 55/143), *P* = 0.014, 0.030. There were no significant difference with SS rates between spontaneous SAE and hepatotropic viruses superinfection groups (*P* = 0.314).

Spontaneous survivors (36.9 ± 17.3 years) tended to be younger than patients who were not spontaneous survivors whether in non-HBV (36.9 ± 17.3 versus 42.2 ± 18.7, *P* = 0.035) or HBV associated liver failure (38.6 ± 11.0 versus 43.6 ± 11.6, *P* = 0.000). There was no significant difference with SS rate between male (28.8 %, 36/125) and female (27.9 %, 39/140) patients (*P* = 0.865) in non-HBV associated liver failure. In HBV associated liver failure, SS rate was higher in female (35.4 %, 150/424) than 31.1 % (771/2482) of male patients, but having no statistically significant difference (*P* = 0.08). Table 3 showed the relationship of outcome with sex and age of patients in HBV associated liver failure. For male patients, there were significant difference with SS rates among different age groups (*P* = 0.000), which SS rate was the highest in those aged 20–24 years (63.1 %, 53/84), then decreased progressively with increasing age. For male, SS rate was significantly higher in patients aged 30–34 years (41.8 %) than 30.3 % of aged 35–39 years group (*P* = 0.001). For female, no significant difference was observed with SS rates among different age groups below 55 years (*P* = 0.564), and also among different age groups over 55 years (*P* = 0.322), but SS rate was significantly higher in patients aged <55 years group (42.5 %, 131/308) than 16.4 % (19/116) of aged ≥55 years group (*P* = 0.000). SS rates were higher in male than in female patients aged <25 years [59.8 % (73/122) versus 43.5 % (10/23)] and ≥55 years [18.7 % (65/347) versus 16.4 % (19/116)] group, though having no statistically significant difference (*P* = 0.154, 0.569). However, SS rates of female were all higher than those of male patients in each group of ages 25 to 54 years old. During ages of 40 to 44 years, SS rate was significantly higher in female (50.9 %, 29/57) than 27.6 % (118/427) of male patients, *P* = 0.001.

## Discussion

The current study is the first systematic investigation on the presumed etiologies or trigger factors and prognosis of patients with liver failure in Southwest China. Although all data were collected only in our hospital, we considered that our results were able to reflect the real-life characteristics of liver failure in Southwest China because that 98.3 % enrolled patients were not only from Chongqing city, but also from other five provinces of Southwest China. Our results showed that ACLF was the predominant clinical type of liver failure in Southwest China accounting for 2922 out of 3171 cases (92.1 %), being similar with other results reported in other area of China [12]. Among 1343 hospitalized patients with cirrhosis from Europe, the prevalence of ACLF was 30.9 % [13]. However, there was no data about the percentage of ACLF in liver failure from developed countries.

The etiologies or trigger factors of liver failure are numerous and varied, including primary hepatotropic viruses, secondary hepatotropic viruses, drug, alcohol, metabolic causes, AIH, pregnancy, and so on. In this study, a total of 25 etiologies or trigger factors were identified to be associated with the onset of liver failure, in which the 5 leading causes were HBV, alcohol, drug, withdrawal of NUCs and HEV. But, another 2.68 % patients remained indeterminate etiology after extensive evaluation. However, the contribution of each etiology was different in ALF, SALF and ACLF. The 3 leading causes of ALF and SALF all included single HBV infection, indeterminate and drugs, but the first common cause was single HBV infection in ALF accounting for 46.7 % cases, and indeterminate in SALF accounting for 29.3 % cases. Our result was similar with the report from Japan, but showed a significant difference compared with other reports from western developed countries and other areas of China [14]. According to our investigation, HBV infection absolutely predominated in the etiologies of ACLF accounting for 96.5 % cases in China, which could be significantly different in western developed countries, because that the most common etiology of chronic liver disease was HBV infection in China [2], but was alcoholism in western developed countries [1]. The major etiologic agents responsible for precipitating ACLF are quite distinct in the East and the West. Alcohol and drugs constitute the majority of acute insults in the West, whereas infectious etiologies predominate in the East [9]. In Bangladesh, acute HEV infection was a leading cause of ACLF accounting for 21.7 % (15/69) patients [7]. According to an investigation from Southeast China during the 12 years between 1993 and 2004, an important factor responsible for precipitating HBV associated ACLF was the superinfection of hepatotropic viruses accounting for 37.9 % (107/282) patients, which included HAV (1.4 %), HCV (6.4 %), HDV (1.8 %) and HEV (28.4 %) [9]. The causes were not clear in another 62.1 % patients. Of these patients, the most possible cause was considered to be spontaneous SAE of CHB. Our results showed that the first leading cause of HBV associated ACLF was also spontaneous SAE of CHB accounting for 62.5 % cases, and the second leading cause was alcohol (15.4 %), and the third leading cause was withdrawal of NUCs (5.7 %), and the fourth leading cause was hepatotropic viruses superinfection (5.2 %) during the 13 years between 2000 and 2012. However, the second leading cause of ACLF was hepatotropic viruses superinfection accounting for 17.1 % cases during the 3 years between 2001 and 2003, which was similar with the report from Southeast China. Our results showed that the precipitating factors were the most complex in ACLF, which included 29 different patterns derived from 25 causal agents compared with 15 patterns of SALF and 11 patterns of ALF. Otherwise, 36.7 % patients had at least two precipitating factors in ACLF, but only 6.7 % in ALF and 1.7 % in SALF.

During the 13 year period between 2000 and 2012, the prevalence of HAV, HCV, HDV and HEV infection was a gradually declined trend among patients with liver failure of Southwest China, which was all lower than 2.5 % after 2006. These results indicate that hepatotropic viruses other than HBV have not been the common etiology of liver failure in current Southwest China. The explanations for this included that safe blood products, hepatitis A and E vaccines were widely used in China.

Antiviral therapy with NUCs has been confirmed to be effective in preventing the disease progression of chronic hepatitis B, but hepatitis flares related to NUCs therapy has become an urgent clinical problem that we have to face. Several reports showed that 10–20 % of patients experienced withdrawal flares of hepatitis B after cessation of NUCs therapy [15, 16]. Hepatitis flares due to HBV resistance are also common during NUCs therapy [17]. Several case reports have been published about ACLF induced by acute hepatitis flares due to the withdrawal or resistance of NUCs. However, there was no data about the contribution of antiviral therapy (AVT) related hepatitis flare in the development of ACLF. Our results showed that the percentages of patients with ACLF due to withdrawal and resistance of NUCs were gradually increased from none of 142 cases in 2000 up to 9.4 % and 2.2 % in 2012. During the 13 year period, the total contributions to the etiologies of ACLF were 7.3 % by ART related hepatitis flare, 6.2 % by superinfection of hepatotropic viruses, and 3.2 % by HBV plus drugs. Importantly, the percentages of patients with ART-ACLF were markedly increased from 0 % in 2000 up to 11.5 % in 2012, while the percentages of ACLF associated superinfection were rapidly decreased from peak 19.3 % in 2002 down to 2.5 % in 2012. During the 6 year period between 2007 and 2012, AVT related hepatitis flares (10.1 %) have become the third leading cause of ACLF in place of superinfection (2.6 %) and HBV plus drugs (3.0 %). These finding firstly indicated that AVT related hepatitis flares played an important role in the development of HBV associated ACLF. So, doctors should pay more attention to the management of CHB patients who received antiviral therapy. Otherwise, our data showed that 61 (2.1 %) cases of ACLF were associated with the reactivation of HBV (59 cases) or HCV (2 cases) induced by steroids use. China is a region of high prevalence of HBV infection, and the carrier rate of HBsAg in adults is more than 8 % [17]. Many cases with known or unknown chronic HBV/HCV infection need to receive an immunosuppressive treatment because of suffering from other diseases. However, the reactivation of HBV or HCV is common following immunosuppressive treatment and is associated with a high mortality [18, 19]. Our results also showed that steroids use induced ACLF cases had a particularly dismal SS rate of 11.1 %. Therefore, doctors must highlight the screening for HBV and HCV infection in all candidates of immunosuppressive treatment, and an effective antiviral therapy should be taken for patients with HBV or HCV infection before receiving an immunosuppressive treatment.

In this study, our results showed that ACLF was more common in elderly male population compared to ALF and SALF. The explanations for this included that 96.5 % ACLF cases were associated with chronic HBV infection in this study, and elderly male tended to take alcohol in China. Compared to antituberculosis drugs induced liver failure, Chinese herbs induced liver failure tended to be elderly female population. The possible reason is that elderly females are more likely to take Chinese herbs for treating diseases in China. Especially importantly, our results showed that the development of liver failure was more frequent in male HBV carriers than in female carriers. The ratio of male to female in HBV related liver failure was 5.9:1, which was significantly higher than 1.5:1 (male 8.6 % versus female 5.7 %) of HBsAg in China [20]. This sexual dimorphism was also observed in HBV related cirrhosis and primary hepatocellular carcinoma [21]. Otherwise, male patients tended to be younger than female cases in HBV associated liver failure. 81.1 % male patients were aged 25–55 years, but 67.2 % female patients were aged 25–55 years, the ratio of male to female was 7.1:1. These findings indicated that male HBsAg carriers were at higher risk for liver failure compared to female carriers. The possible reason may be due, at least in part, to lower production of estrogen and a reduced response to the action of estrogen [22]. Estrogen is a potent endogenous antioxidant which attenuates induction of redox sensitive transcription factors and hepatocyte apoptosis by inhibiting generation of reactive oxygen species, Furthermore, estradiol can prevents the autocrine loop of reactive oxygen species (ROS) by suppressing NADH/NADPH oxidase activity, and has a cytoprotective effect against hepatocyte injury. [23].

Our results showed that the average 3-month SS rate of liver failure was only 22.2 % in 6 years between 2000 and 2005, but up to 32.4 % in 4 years between 2006 and 2009, and reached to 41.1 % in 3 years between 2010 and 2012. These results indicated that our ability in treating liver failure has significantly improved. The trend toward increased SS in the last 30 years was considered to be primarily attributed to improved critical care management in developed countries [24]. In China, the improved SS may be associated with the early introduction of antiviral therapy for hepatitis B, steroids use, plasma exchange and hemodiafiltration. Of course, it was also attributed to the effective prevention and treatment of complications, and improved critical care management.

According to the diagnostic criteria of ALF, SALF and ACLF in China, our results showed that SS rate of ALF cases was significantly lower than SALF and ACLF cases. However, SS rate of ALF cases was significantly higher than SALF and ACLF cases with HE. For SALF and ACLF, SS rate was significantly higher in cases without HE than in cases with HE. These findings suggested that the onset of HE was an important indicator of worse prognosis among patients with liver failure.

As all known, the prognosis of liver failure also depends on their etiologies. Patients with acetaminophen toxicity had the best outcome in developed countries [25]. Our results also showed that the SS rate was also related to the cause of liver failure, which the highest SS rate was AFLP, and the lowest was patients infected by HIV combined with HBV or HCV. Our results showed that patients with HBV related ACLF induced by alcohol, drugs, NUCs withdrawal and HBV resistance to NUCs had significantly lower SS rate compared with ACLF cases induced by spontaneous SAE of CHB and hepatotropic viruses superinfection, but did not show that SS rate was lower in patients with hepatotropic viruses superinfection than in spontaneous SAE of CHB cases. These findings indicated that alcohol, drugs, NUCs withdrawal and HBV resistance responsible for precipitating liver failure had more severely harmful effects on chronic HBV carriers compared with hepatotropic virus superinfection. So, it is especially important for chronic HBV carriers to avoid intake of alcohol and hepatotoxic drugs, and for CHB patients with NUCs treatment to avoid hepatitis flares due to NUCs withdrawal and HBV resistance.

Older age has been widely confirmed to be associated with the severity of various liver diseases and a poor prognosis of ALF in many studies. Regev and Schiff reported 3–5-fold increases in deaths due to liver diseases in those over 65 years of age versus those under 45 years [26]. From India, Dhiman et al. reported that age greater than 50 years was an independent predictor of outcome in patients with viral ALF [27]. Our data showed that spontaneous survivors tended to be younger than patients who died or received a liver transplant whether in non-HBV or in HBV related liver failure. In male patients with HBV related liver failure aged ≥25 years, SS rates decreased progressively with increasing age, and a statistically significant difference was observed firstly between age 30 to 34 years and 35 to 39 years group. In female cases of HBV related liver failure, SS rates were only observed to be significantly higher in age <55 years group than in age ≥55 years group. These findings also indicated that older age was associated with a poor prognosis of liver failure, especially in male patients with HBV related liver failure. The mortality of HBV related liver failure increased markedly with increasing age ≥35 years in males and ≥55 years in females.

As the incidence of HBV related diseases being unequal between males and females, this sexual dimorphism was also observed in the outcome of HBV related diseases. Szpakowski JL, et al. reported that HBV-related mortality was four times more common in males than in females [28] (Causes of death in patients with hepatitis B: a natural history cohort study in the United States). Our results showed that SS rate was not lower in male than in female patients for non-HBV related liver failure, but was lower in male than in female patients for HBV related liver failure although having not reached a statistically significant difference. However, the results of subgroup analysis based on different age groups showed that SS rates were all lower in male than in female patients with HBV related liver failure among each age group from 25 to 54 years old, and reached a statistically significant difference in cases of ages 40 to 44 years old. Interestingly, SS rates of male patients were higher than those of female patients in age <25 years (59.8 % versus 43.5 %) and ≥55 years (18.7 % versus 16.4 %) group, though having no statistically significant difference. These findings further indicated that male was also associated with a poor prognosis of HBV related liver failure in the middle-aged patients.

## Conclusions

In conclusion, etiologies of liver failure were numerous and varied during the 13 year period between 2000 and 2012 in southwest China. Recent years, there have been some new changes in clinical and epidemiological characteristics of liver failure, in practical work, knowing these are helpful to judge the diagnosis and prognosis of liver failure, and then take positive and effective measures so as to reduce the mortality of liver failure. HBV was the most leading cause of liver failure, and absolutely predominated especially in the etiologies of ACLF. AVT related hepatitis B flares increased progressively, and had become the third leading cause of ACLF in place of hepatotropic viruses superinfection and drugs. The prognosis of liver failure remained very poor, but had markedly improved in recently 3 years. ACLF patients induced by alcohol, drugs, NUCs withdrawal and HBV resistance had worse prognosis than those induced by spontaneous SAE of CHB. Liver failure induced by reactivation of HBV or HCV due to steroids use was not uncommon, and had an extremely high mortality. Male was at higher risk for liver failure and death compared to female in the middle-aged HBsAg carriers. Older age was associated with a poor prognosis of liver failure, especially in male patients with HBV related liver failure.

## Abbreviations

ACLF: acute-on-chronic liver failure
AFLP: acute fatty liver of pregnancy
AIH: autoimmune hepatitis
ALD: alcoholic liver disease
ALF: acute liver failure
AVT-ACLF: antiviral therapy related ACLF
CMV: cytomegalovirus
DILI: drug induced liver injury
EBV: Epstein-Barr virus
HAV: hepatitis A virus
HBV: hepatitis B virus
HCV: hepatitis C virus
HDV: hepatitis D virus
HE: hepatic encephalopathy
HEV: hepatitis E virus
HIV: human immunodeficiency virus
HSV: herpes simplex virus
NUCs: nucleos (t)ide analogues
PTA: prothrombin activity
SAE: severe acute exacerbation
SALF: sub-acute liver failure
SS: spontaneous survival
S-SAE: spontaneous severe acute exacerbation
T-Bil: total bilirubin
